# Evaluating Kaposi Sarcoma in Kidney Transplant Patients: A Systematic Review and Meta-Analysis

**DOI:** 10.7759/cureus.52527

**Published:** 2024-01-18

**Authors:** Sakditad Saowapa, Natchaya Polpichai, Pharit Siladech, Chalothorn Wannaphut, Manasawee Tanariyakul, Phuuwadith Wattanachayakul, Pakin Lalitnithi

**Affiliations:** 1 Internal Medicine, Texas Tech University Health Sciences Center, Lubbock, USA; 2 Internal Medicine, Weiss Memorial Hospital, Chicago, USA; 3 Internal Medicine, Ramathibodi Hospital, Bangkok, THA; 4 Internal Medicine, University of Hawaii John A. Burns School of Medicine, Honolulu, USA; 5 Internal Medicine, Einstein Medical Center Philadelphia, Philadelphia, USA; 6 Internal Medicine, St. Elizabeth's Medical Center, Boston, USA

**Keywords:** kaposi sarcoma, renal transplantation, immunosuppressant, cancer epidemiology, cancer incidence, kaposi sarcoma hiv negative

## Abstract

Kaposi's sarcoma (KS) is a malignancy that commonly appears as lesions on the skin or mucosal surfaces but can also develop in other organs. This cancer is usually caused by the human herpesvirus 8 (HHV-8), recently known as Kaposi’s sarcoma-associated herpesvirus (KSHV). KS is rare in the general population but can develop in kidney transplant recipients with varying incidence due to immunocompromised status from immunosuppression.

The main aim of the present systematic review was to identify the prevalence and treatment of KS in kidney transplant patients. PubMed, Cochrane Library, and Google Scholar databases were searched for studies until October 2023. Full-text studies with similar research objectives were included, while non-English articles, reviews, case reports, ongoing clinical trials, and studies evaluating KS in HIV patients or after other solid organ transplants were excluded. All studies were observational; therefore, methodological quality was assessed using the Newcastle-Ottawa Scale. The statistical analyses were performed with the Comprehensive Meta-Analysis (CMA) software (Biostat, Inc. Englewood, NJ).

The pooled analysis from the 15 studies included showed that KS develops in 1.5% of kidney transplant recipients and is more prevalent in African (1.7%) and Middle Eastern (1.7%) recipients than in Western recipients (0.07%). KS was also significantly more prevalent among male recipients than female recipients (OR: 2.36; p < 0.0001). Additionally, cyclosporine-based immunosuppression accounts for most KS incidences (79.6%) compared to azathioprine-based immunosuppression (28.2%). Furthermore, reduction or withdrawal of immunosuppression alone resulted in 47.8% KS complete remissions.

Post-kidney transplantation KS is more frequent among males and patients of Middle Eastern and African origin. However, the gender difference may be attributed to most patients undergoing kidney transplants being male. Therefore, if gender balance is considered in future studies, then the difference might be insignificant. Based on our results, we can concur that the mainstay treatment for post-transplant KS is reduction or withdrawal of immunosuppression. However, the patients should be closely monitored to avoid KS recurrence and kidney rejection. Furthermore, there is an increased risk for KS with the use of cyclosporine-based immunosuppression. However, this does not mean that the withdrawal of this immunosuppression agent might result in improved KS outcomes because the withdrawal of azathioprine with or without cyclosporine reduction has also led to improved outcomes.

## Introduction and background

Kaposi's sarcoma (KS) first gained notoriety in 1872 following a description by physician and dermatologist Moritz Kaposi [1]. The disease has been described as a multi-focal neoplasm originating from the endothelial region, with a predominance witnessed in elderly males of Eastern European or Mediterranean descent [2]. According to Kaposi, the disease had a devastating mortality rate of at least two years following infection [1]. Over time, the landscape of KS has evolved, especially due to growing concern in the organ transplantation domains, where the triumph of grant survival is juxtaposed with the challenges and perils of opportunistic infections and possible neoplasms [3].

Antman and Chang describe KS of Kaposi’s sarcoma-associated herpesvirus (KSHV) as an “indolent agio-proliferative spindle-cell tumor derived from human herpes virus type-8 infected-immune and endothelial cells” [4]. Therefore, the human herpesvirus type-8 (HHV-8) is described as the major causative agent of general patients with KS, presenting in over 95% of all diagnosed cases [4]. However, Raeisi et al. observe that the etiopathogeneses of the KS malignancy are still not fully understood due to their complexities [5]. The intricate link between the HHV-8 virus and the immune system has been articulated as the key mechanism in the genesis of KS, with the malignancy observed in immunosuppressed patients as a result of acquired immunodeficiency syndrome (AIDS) infection or organ transplants [6]. This malignancy is characterized by purple-reddish or bluish, non-painful, non-itchy plaques, macules, and papules, which are highly vascularized and can easily ulcerate and bleed [7]. Moreover, KS is not exclusively restricted to the skin but is expressed also in the bodily mucosal surfaces such as the lungs, the gastrointestinal tract, and the lymphoid tissues.

A kidney transplant requires a delicate balance between immunosuppression to decrease the likelihood of graft rejection while controlling for increased susceptibility to viral infection at the same time [7]. Euvrard et al. argue that the incidence of KS is closely associated with the duration and intensity of immunosuppression among kidney transplant patients, especially in patients where HHV-8 serology is present pre-transplantation [8]. This incidence has been documented to occur from a few weeks post transplantation to 18 years, with most cases reported after 13 months [8].

Furthermore, Raeisi et al. observe that the long-term utilization of immunosuppressive agents in organ transfers in preventing allograft rejection is associated with a significant (100%) increase in malignancy in these patients compared to the general population. However, the epidemiology and prevalence rate of transplant-associated malignancies is highly divergent depending on the geographical locations. KS exists in four clinical forms; the classic KS predominates among elderly males of Mediterranean and Eastern European affiliation, largely manifested in the lower extremities [6]. Secondly, the endemic African KS is generalized in the lymph nodes and occurs in children. Thirdly, HIV-associated KS occurs mainly in patients not in highly active antiretroviral therapy, occurring mainly on the skin and internal organs, and finally, the iatrogenic KS variation in immunosuppressed patients [6].

In the context of this systematic review and meta-analysis, our focus lies largely on the iatrogenic KS with a male-to-female ratio of 3:1, and present in over 5% of transplant patients who are prone to develop a de novo malignancy [9]. As we navigate the realms of kidney transplantation and oncology, it becomes apparent that the landscape is not only characterized by the clinical manifestations of KS but also by the therapeutic conundrums that clinicians face in its wake. With a lens focused on both the molecular intricacies of KS development and the practicalities of patient care, this systematic review aims to unravel the complexities surrounding this malignancy in kidney transplantation. By exploring the historical context, current challenges, and future directions in research, we seek to contribute to the broader understanding of KS, ultimately guiding the way toward more effective prevention, diagnosis, and treatment strategies for kidney transplant recipients. The primary objective for performing this systematic review and meta-analysis was largely cemented on analyzing the prevalence and treatment of KS in kidney transplant patients.

## Review

Materials and methods

Research Design

The systematic review and meta-analysis strictly followed the guidelines outlined in the Preferred Reporting Items for Systematic Reviews and Meta-Analyses (PRISMA) 2018 by Tricco et al. [10].

Literature Search Strategy and Screening

In the October-November 2023 period, an extensive literature search was systematically conducted using key phrases such as renal transplant, Kaposi sarcoma, liver cancer, immunosuppression, human herpesvirus-8 (HHV-8), and Kaposi's sarcoma-associated herpesvirus (KSHV) to obtain pertinent data addressing the research question. Utilizing medical databases, a predefined search string was generated from relevant keywords incorporating Medical Subject Heading (MeSH) terms. The search strategy incorporated Boolean operators, truncations, and filled tags to optimize the search process, as articulated by Russell-Rose and Chamberlain [11]. The National Library of Medicine - National Institutes of Health library, housing medically significant journal databases such as PubMed and Clinical Trials, served as the primary literature source. Additionally, the Cochrane Library, Web of Science (WOS), and MEDLINE databases were thoroughly examined to identify literature relevant to the research question.

This comprehensive literature search aimed to identify approved, peer-reviewed, and published papers suitable for inclusion in the subsequent systematic review and meta-analysis. Additionally, the study and analysis sought to disclose supplementary data sources obtained by scrutinizing the references of retrieved articles, aiming to identify additional literature for incorporation into the meta-analysis. Furthermore, a meticulous exploration of peer-reviewed yet unpublished grey literature was conducted through manual searches of pertinent green papers submitted to universities and research centers.

**Figure 1:**
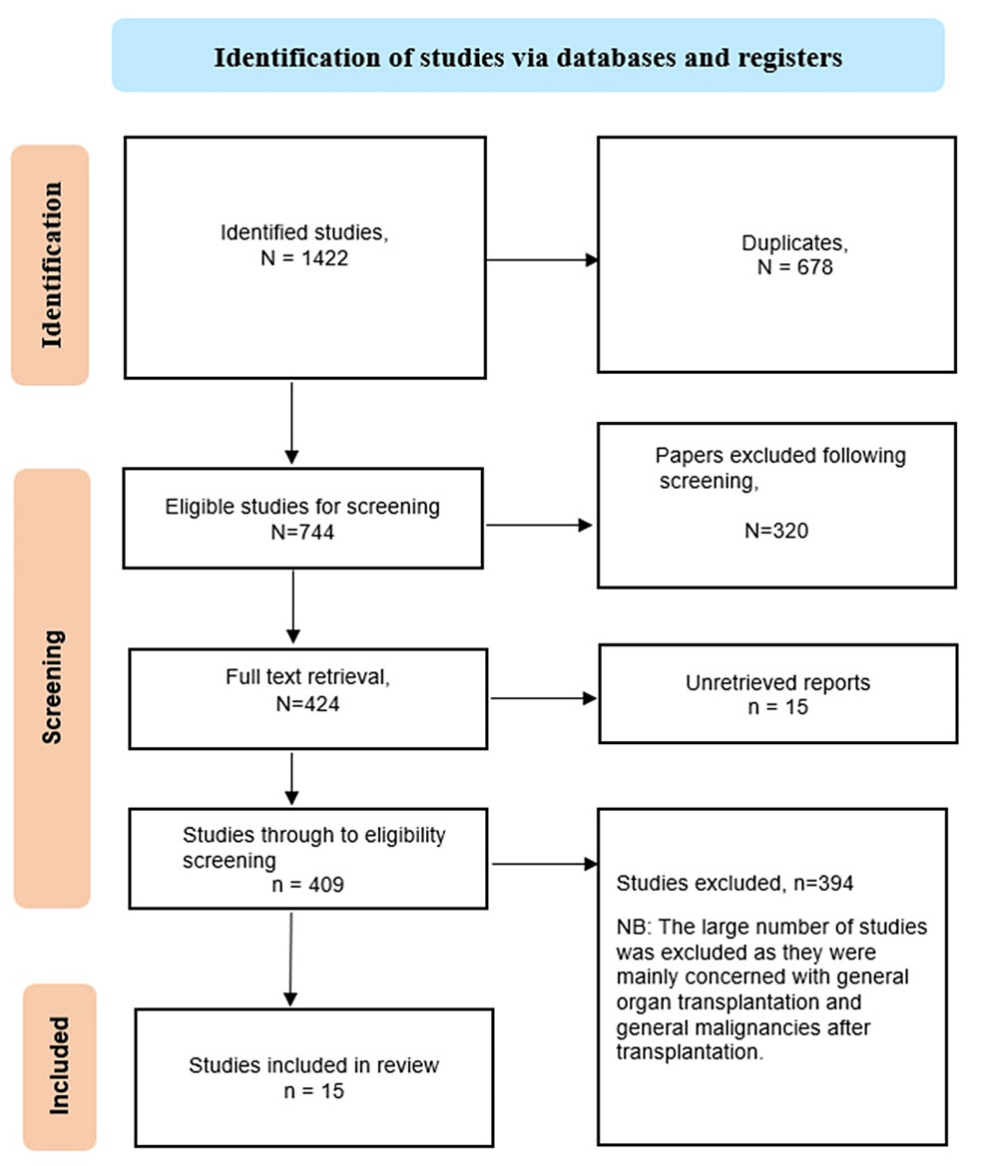
PRISMA flowchart summarizing the search strategy PRISMA: Preferred Reporting Items for Systematic Reviews and Meta-Analyses.

The initial database search yielded a total of 1422 studies. Following meticulous abstract and title scrutiny, 678 duplicate records were removed. Subsequent in-depth examination resulted in the exclusion of 320 studies based on abstract and title assessment. Additionally, 15 studies lacking abstracts were excluded. Further exclusions included 250 studies incompatible with this meta-analysis's objectives, along with those presented as posters and non-English studies. Following these refined screening processes, 100 studies proceeded to the eligibility screening. Subsequently, 44 studies were deemed unsuitable for inclusion in the review. As a result, 15 studies advanced to the quantitative test accuracy assessment for reliability and validity (Figure 1).

Study Selection and Data Collection

After conducting an initial search across the specified databases, our systematic review and meta-analysis engaged the expertise of two independent reviewers to thoroughly examine the identified papers' titles, abstracts, and full texts. The evaluation of full-text analysis for records meeting inclusion criteria involved critical assessment by all authors. Discrepancies in estimates were addressed through collaborative discussions led by the principal researcher, and external consultations with statisticians and professionals in the research field were sought to enhance the depth of insights for the meta-analysis.

The studies identified during the initial database search were meticulously documented in an Excel spreadsheet (Microsoft Corporation, Redmond, WA). To facilitate the screening process against eligibility criteria, a separate spreadsheet was dedicated to categorizing the collected data. The authors collaboratively developed a narrative table that delineated the distinctive characteristics of each study, ensuring a comprehensive and well-organized synthesis of the research findings.

Eligibility Criterion

To further optimize the study selection process, the authors established eligibility criteria to guarantee the inclusion of only the most pertinent articles and studies in the meta-analysis using PICOs (Populations, Intervention, Comparisons, and Outcomes). Consequently, the systematic review and meta-analysis incorporated only those studies that adhered to the criteria detailed below.

Inclusion Criteria

Population (P): Only published peer-reviewed articles on patients diagnosed with KS following kidney transplants, specifically kidney transplants, were included in this systematic review and meta-analysis. No filters on age, gender, or ethnic variations were considered for the populations.

Interventions (I): Only published, peer-reviewed manuscripts detailing KS treatment in kidney recipients were included in the review. No parameters to the kind of immunosuppression utilized were used; however, the review found it helpful to restrict immunosuppressive intervention to cyclosporine and azathioprine to achieve a more consolidated treatment regime.

Comparisons (C): Studies comparing the outcomes between the withdrawal or reduction of the immunosuppressive regimen, the type of immunosuppressive regimen, and the overall or partial remission of KS were included in the review. Moreover, studies detailing a comparison between male and female kidney transplant recipients were also included in the review.

Outcomes (O): Studies reporting results following the withdrawal or partial withdrawal of treatment were included in the study.

Exclusion Criteria

Studies were excluded based on the following criteria: (1) studies published in a language other than English. Language restriction was essential in avoiding the translation of scientific terms; (2) secondary studies, including posters and case series; (3) studies evaluating general organ transplantation and general malignancies after transplantation.

Data Analysis and Quality Appraisal

The Comprehensive Meta-Analysis (CMA) tool (Biostat, Inc. Englewood, NJ) was utilized as the primary data analysis tool. Data were analyzed in terms of event rates (ER) using a 95% confidence interval (CI). P-values lower than 0.05 (p < 0.05) were considered statistically significant. Based on the PRISMA guidelines, the meta-analysis adopted the National Heart, Lung, and Blood Institute (NHLBI) quality assessment tool, which was utilized based on the retrospective nature of the studies included in the meta-analysis [12].

Results

Study Characteristics

Most of the studies were retrospective studies with study years ranging between five and 10 years of investigation, apart from a cross-sectional analysis conducted by Shahbazian [13] (Table 1). A total of 323 participants from the study were diagnosed with KS from a total population sample of 177,532 across the 15 included studies. Most of the studies were based in Europe (Italy, Portugal, Spain, and Greece). Four of these studies were based in Asia (Iran and Saudi Arabia) while one study based in the USA was reported. Moreover, four studies were conducted in Africa (South Africa, Tunisia, and Egypt). The overall mean age ranged from 42 (IQR: 30.5-50.2) years. The time to develop KS was more than or equal to eight months following treatment initiation.

**Table 1:** Characteristics of included studies NR: not reported; SD: standard deviation; IQR: interquartile range; KS: Kaposi sarcoma; HHV-8: human herpesvirus-8; HHV: human herpesvirus; sVCA: small viral capsid antigen. Most of the studies were retrospective studies with study years ranging between 5 and 10 years of investigation, apart from a cross-sectional analysis conducted by Qunibi et al. [25].

Author ID	Study location	Study design	Population (N)	Patients with KS (N)	Complete remission upon reduction or withdrawal of immunosuppression	Mean age/mean ± SD/median (IQR) (years)	Time taken to develop the sarcoma, mean/mean ± SD/median (IQR)/range (months)	Findings
Shahbazian (2004) [13]	Iran	Retrospective	580	14	9	41	8 to 31	KS prevalence in Iran compared to the incidence rates in Western countries. Moreover, KS was rarely characterized by visceral involvement and a poor prognosis. The discontinuation of immunosuppressants azathioprine and cyclosporine was associated with a cessation of skin evolvement and preservation of renal functions.
Margolius et al. (1994) [14]	South Africa	Retrospective	989	5	3	47	18 (8-38)	The complete withdrawal of immunosuppressants such as steroids, azathioprine, and cyclosporine was associated with tumor regression.
El-Agroudy et al. (2003) [15]	Egypt	Retrospective	1400	24	NR	29.8 ± 11.1	33.9 ± 27.2	Early diagnosis and interference were associated with a more favorable prognosis.
Silva et al. (2007) [16]	Portugal	Retrospective	1479	6	NR	44 ± 13	13 to 34	KS was characterized as an aggressive multi-organ malignancy presenting in six patients from July 1983 to September 2006 following kidney transplantation. Conversion to a less aggressive immunosuppressant and chemotherapy were associated with successful malignancy management.
Bencini et al. (1993) [17]	Italy	Retrospective	820	11	NR	35.4 ± 11.7	NR	The administration of immunosuppressants cyclosporine, azathioprine, and methylprednisolone is associated with KS incidence among kidney transplant patients. Moreover, the study affirms that immunosuppression with azathioprine and methylprednisolone was associated with a 500-fold increase in kidney transplant patients, while cyclosporine was associated with a 1000-fold increase in these patients.
Einollahi et al. (2009) [18]	Iran	Retrospective	7939	55	NR	50 ± 11	27 ± 33	The study noted a significantly higher incidence of KS in the country, suggesting a crucial role of genetic predisposition in the incidence of KS. Moreover, the reduction or total withdrawal of immunosuppression was associated with complete remission of the KS.
Gorsane et al. (2016) [19]	Tunisia	Retrospective	568	12	5	33.5 (26-50)	23.3 (3-96)	The annual KS incidence ratio of 2.1%, which was a 0.27% higher difference compared to other European Mediterranean countries, was reported in the study.
Raeisi et al. (2013) [5]	Iran	Retrospective	1487	17	NR	47.8 ± 23.4	18.7 ± 25.2	Among kidney transplant patients, the incidence of KS post-transplantation was significant (P < 0.05), with the highest incidence rates reported among men (58.8%), patients with Cellcept and azathioprine immunosuppressants (70.5% and 29.4%, respectively). Moreover, the presence of HHV serum antibodies is a crucial characteristic of KS incidence in organ transplant patients.
Cahoon et al. (2018) [20]	United States	Retrospective	157,105	91	NR	NR	NR	Due to the administration of immunosuppressive drugs, individuals who have undergone solid organ transplants face a heightened susceptibility to cancers induced by oncogenic viruses, notably KS. This extensive population-based study of transplant recipients revealed that factors possibly correlated with HHV-8 infection or heightened immunosuppression were linked to an elevated risk of KS. Although no particular medications showed a significant association with KS risk, recipients who had undergone recent transplants exhibited a reduced risk of KS, hinting at potential enhancements in drug-prescribing practices.
Lesnoni et al. (1997) [21]	Italy	Retrospective	302	10	4	46.4 ± 9.4	21.1 ± 17.6	Immunosuppression forms the leading factor in KS incidence among kidney transplant patients. KS was highly prevalent in the kidney transplant group compared to other organ transplants. This observation confirms that genetic predisposition was a key issue in KS patients of Italian descent besides viral factors.
Mitxelena et al. (2003) [22]	Spain	Retrospective	1230	6	NR	53.6	10.6 (4-36)	Immunodeficiency is significantly associated with the development of KS among kidney transplant recipients. Reduced immunosuppression was also associated with improvement in cutaneous KS.
Montagnino et al. (1994) [23]	Italy	Retrospective	820	13	8	36.8 ± 11.1	38.7 ± 38.3	KS was associated with multiple skin and mucosal/visceral lesions with heterogeneous clinical manifestations. Immunosuppression withdrawal was associated with remission of the KS with no compromise to the graft function.
Moosa (2005) [24]	South Africa	Retrospective	542	21	NR	42 (27-54)	22.2 (2.9-64.1)	The study finds that KS was equal among males and females with universal skin involvement. Moreover, cyclosporine was not associated with increased KS frequency. Thus, reducing immunosuppression should be the first-line treatment of the malignancy when the disease is limited to the skin.
Qunibi et al. (1998) [25]	Saudi Arabia	Cross-sectional	263	14	3	41.8 ± 11.9	NR	The study reports a higher incidence of KS in kidney transplantation patients with HHV-8 p40 and sVCA antibodies.
Zavos et al. (2014) [26]	Greece	Retrospective	2008	24	NR	47.7	33.7 (3-138)	The reduction or overall withdrawal of immunosuppressants was associated with remission of the KS. Generally, the prognosis of patients where the KS was limited to the skin was favorable. In contrast, visceral involvement was associated with a high mortality rate among kidney transplant patients diagnosed with KS.

Kaposi Sarcoma Prevalence According to the Region

The analysis stratified the prevalence of KS in terms of geographical position, with significant strata being Africa, Arabian, and Western. However, the Arabian and Western stratifications mainly comprised European studies [16,17,21-23,26] and a single study from the United States [20]. Meanwhile, the Arabian studies included Asian countries, particularly Iran and Saudi Arabia [5,13,18,25]. The African strata were primarily composed of Mediterranean countries, north of the African continent, Egypt, and Tunisia [15,19], except for a study conducted in South Africa [24].

**Figure 2:**
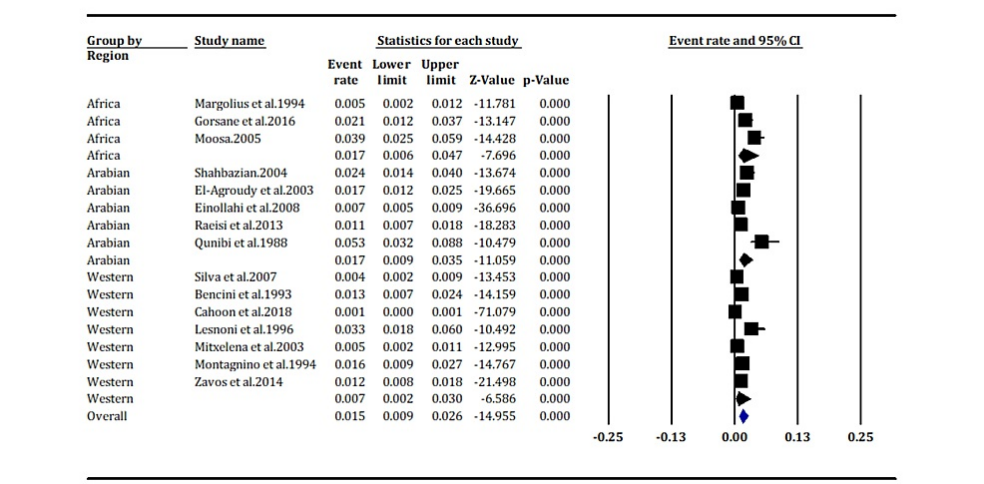
The prevalence of Kaposi Sarcoma according to region (overall p-value < 0.05) Margolius et al. (1994) [14]; Gorsane et al. (2016) [19]; Moosa (2005) [24]; Shahbazian (2004) [13]; El-Agroudy et al. (2003) [15]; Einollahi et al. (2008) [18]; Raeisi et al. (2013) [5]; Qunibi et al. (1998) [25]; Silva et al. (2007) [16]; Bencini et al. (1993) [17]; Cahoon et al. (2018) [20]; Lesnoni et al. (1996) [21]; Mitxelena et al. (2003) [22]; Montagnino et al. (1994) [23]; Zavos et al. (2014) [26].

In examining different regional stratifications, this analysis disclosed pertinent information regarding the prevalence of KS. The African stratification exhibited an event rate of 1.7% with a 95% CI of 0.6% to 4.7% (Figure 2). Despite suggesting a plausible association between KS prevalence and African stratification, the association was deemed nonsignificant with a p-value of 0.000. Conversely, the Arabian stratification demonstrated an event rate of 1.7 (95% CI: 0.9%, 3.5%) and a notably low Z-score of -11.059, indicating an extremely low prevalence of KS. The confluence of a low prevalence estimate, a narrow CI, and a significantly negative Z-score provides a high degree of confidence in the assertion that the prevalence of KS in Arabian countries is distinctly low and substantially divergent from the anticipated average.

The Western stratification observed an event rate of 0.7% (95% CI: 0.02%, 3.0%) (Figure 2). The markedly negative Z-score suggests a statistically significant result, indicating that the observed prevalence is considerably lower than expected based on the mean. Similar to the African and Arabian stratifications, the results were significant (p-value < 0.05) (Figure 2).

Kaposi Sarcoma Prevalence in Male Versus Female Recipients

**Figure 3:**
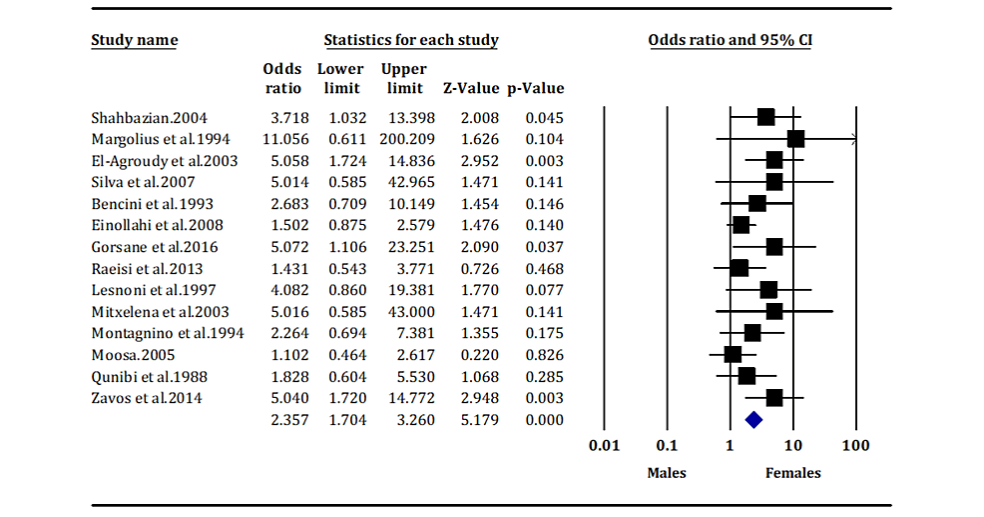
The prevalence of Kaposi sarcoma in male vs. female kidney transplant recipients (overall p-value < 0.05) Margolius et al. (1994) [14]; Gorsane et al. (2016) [19]; Moosa (2005) [24]; Shahbazian (2004) [13]; El-Agroudy et al. (2003) [15]; Einollahi et al. (2008) [18]; Raeisi et al. (2013) [5]; Qunibi et al. (1998) [25]; Silva et al. (2007) [16]; Bencini et al. (1993) [17]; Cahoon et al. (2018) [20]; Lesnoni et al. (1996) [21]; Mitxelena et al. (2003) [22]; Montagnino et al. (1994) [23]; Zavos et al. (2014) [26].

Head-to-head comparison of KS incidence according to gender shows that male kidney transplant recipients are more likely to develop KS than female recipients (OR: 2.357; 95% CI: 1.704, 3.260) (Figure 3).

Incidence of Kaposi Sarcoma in Patients Receiving Cyclosporine-Based Immunosuppression

**Figure 4:**
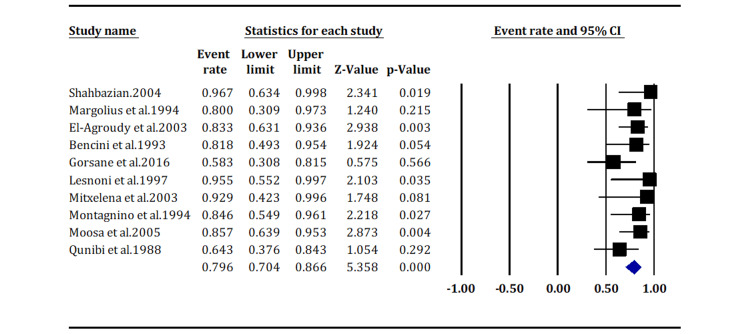
Incidence of Kaposi sarcoma in patients receiving cyclosporine-based immunosuppression (overall p-value < 0.05) Margolius et al. (1994) [14]; Gorsane et al. (2016) [19]; Moosa (2005) [24]; Shahbazian (2004) [13]; El-Agroudy et al. (2003) [15]; Qunibi et al. (1998) [25]; Bencini et al. (1993) [17]; Lesnoni et al. (1997) [21]; Mitxelena et al. (2003) [22]; Montagnino et al. (1994) [23].

The analysis shows that cyclosporine-based immunosuppression is associated with a high incidence of KS after kidney transplantation, with an event rate of 79.6% (95% CI: 70.4, 86.6) (Figure 4).

Incidence of Kaposi Sarcoma in Patients Receiving Azathioprine-Based Immunosuppression

**Figure 5:**
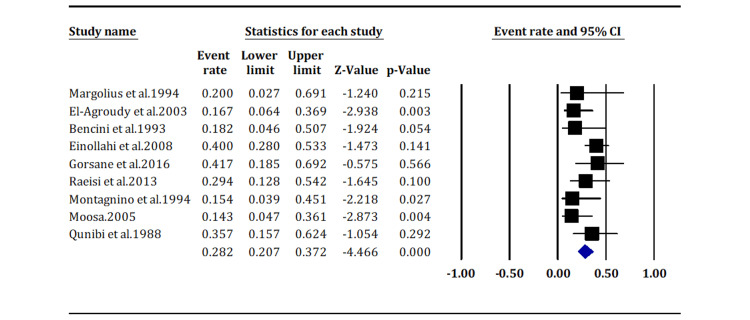
Incidence of Kaposi sarcoma in patients receiving azathioprine-based immunosuppression (pooled overall p-value < 0.05) Margolius et al. (1994) [14]; Gorsane et al. (2016) [19]; Moosa (2005) [24]; El-Agroudy et al. (2003) [15]; Einollahi et al. (2008) [18]; Raeisi et al. (2013) [5]; Qunibi et al. (1998) [25]; Bencini et al. (1993) [17]; Montagnino et al. (1994) [23].

Azathioprine-based immunosuppression without cyclosporine is associated with a low KS prevalence in kidney transplant patients, with an event rate of 28.2% (95% CI: 20.7%, 37.2%) (Figure 5).

In summary, these findings strongly suggest a significant association between azathioprine-based immunosuppression and the incidence of KS, with a relatively precise estimate and high statistical confidence in the observed results.

Impact of Immunosuppression Reduction or Withdrawal on Complete Remission

**Figure 6:**
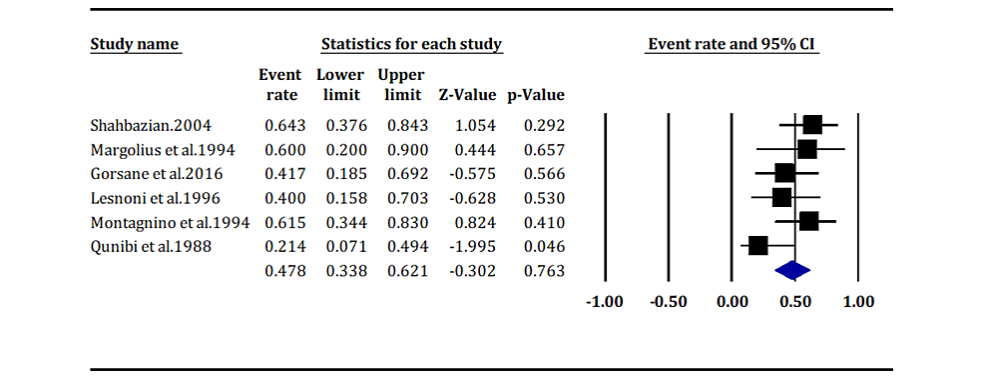
Impact of immunosuppression reduction or withdrawal on complete remission (pooled overall p-value = 0.763) Shahbazian et al. (2004) [13]; Margolius et al. (1994) [14]; Gorsane et al. (2016) [19]; Lesnoni et al. (1997) [21]; Montagnino et al. (1994) [23]; Qunibi et al. (1998) [25].

Withdrawal or reduction in immunosuppression alone results in completion remission in 47.8% of patients with KS, with an events rate of 47.8 (95% CI: 33.8, 62.1) (Figure 6). However, the impact was statistically insignificant (p = 0.763).

Discussion

Kidney transplantation stands as a beacon of hope for individuals with end-stage kidney disease, offering the promise of improved quality of life and extended survival. However, kidney transplantation's success hinges on the delicate equilibrium between preventing graft rejection and minimizing the risk of opportunistic infections and malignancies. KS has been characterized as a critical adversary post-kidney transplantation. According to Raeisi et al. [5], KS incidence peaks during the first years following transplantation, with most cases diagnosed within the first two years following kidney transplantation [5]. Based on findings from the analysis of 15 studies, the time taken was averaged at two years and one month following the kidney allograft placement, which collaborates with observations by Bouwes et al. [27] and Mbulaiteye et al. [28].

The incidence of KS among kidney transplantation patients was stratified into three categories: African, Arabian, and Western, based on evidence from observed data. One fundamental similarity from the meta-analysis of evidenced data showed a nonsignificant association between KS and either geographical location where p = 0.00. This observation can be explained by the type of study design adopted by most of the studies, a retrospective study design that presents several risks, as depicted by Chalmers in 1991 [29]. However, a plausible association between KS and kidney transplantation was noted among the African stratification, as referenced in studies by Sabeel et al. [30]. A reported 13.3% incidence rate was reported in kidney transplantation patients.

Moreover, the Arabian stratification observed a significantly low prevalence rate of KS among kidney transplant patients, contrary to findings by Qunibi et al. [25] and Duman et al. [31]. However, of all diagnosed malignancies following organ, especially kidney transplantation in Saudi Arabia, Iran, and Turkey, KS was the most documented [31-33]. Therefore, there is a need for further studies exploring the prevalence of KS in these regions. In the Western stratification, comprised of predominantly European studies and a US study, the majority of KS was high and significant. This observation affirms previous evidence noting the likelihood of genetic predisposition to KS.

According to Ablashi et al., the prevalence of seropositive IgG to HHV-8 in the healthy population is highest, reaching around 40%, among black South African blood donors and patients with cancers other than KS, where the seroprevalence is 32%. In countries surrounding the Mediterranean Sea, the average seroprevalence is 10%. Conversely, in northern Europe, Southeast Asia, and the Caribbean, seroprevalence falls within the range of 2% to 4%. The data on seropositive prevalence are consistent with the prevalence of KS in kidney transplant patients, suggesting that seropositive populations may be predisposed to developing KS, possibly due to the persistent latent virus in seropositive patients [32].

Post-transplant malignancies are very difficult to treat, mainly as the risk of mortality from the said malignancy is weighed against the risk of graft failure, which, in all cases, is catastrophic for the patient [5]. According to evidence presented by the meta-analysis, the presence of immunosuppressants was associated with the incidence of KS in kidney transplantation patients with cyclosporine and azathioprine immunosuppressants related to a significant incidence of KS in kidney transplantation patients [2]. Research evidence in KS malignancy has documented favorable outcomes, including complete remission, associated with the reduction or overall withdrawal of immunosuppression [5]. This observation aligns with evidence from this meta-analysis showing a strong, despite insignificant, association between KS incidence and total remission after all immunosuppression withdrawals. This reduction of immunosuppression allows the immune system the much-needed time to reduce the viral replication of the disease. Thus, new therapeutic interventions such as antiviral agents are promising options for KS patients following organ transplantation, which need further investigation to gauge their overall efficacy and effectiveness [34-36].

Limitations

The review cites several limitations with the study designs of the included studies, a significant grey area in presenting conclusive evidence of the prevalence of KS. The retrospective study design adopted by most of the included studies is associated with considerable heterogeneity issues regarding symptom reporting, age of incidence and diagnosis, and treatment options. Secondly, the limited number of studies reporting the incidence of KS from 2019 to date hampers our pursuit of presenting updated prevalence rates of KS, especially in the Eastern Mediterranean region. Thirdly, no case follows is represented in the studies; this observation, paired with the significantly low number of identified studies, asserts our recommendations for longitudinal, randomized studies investigating the prevalence of KS.

## Conclusions

The cornerstone of kidney transplantation lies in the modulation of the immune system to foster graft acceptance. However, this intentional dampening of immune responses renders transplant recipients susceptible to a spectrum of complications, including viral-related cancers. As the immune system undergoes modification to accommodate the transplanted organ, the risk landscape shifts, with a notable increase in susceptibility to viral-related cancers. Kaposi's sarcoma, intricately linked to HHV-8, exemplifies the intricate dance between immunosuppression and oncogenesis. Unraveling the nuances of this interplay is crucial for developing targeted prevention and management strategies tailored to the unique needs of kidney transplant recipients.

Further exploration into the molecular and genetic factors influencing KS development can provide insights into the underlying mechanisms, with more in-depth investigations into genetic susceptibility and viral interactions unveiling novel therapeutic targets. Meanwhile, further research into the molecular and genetic factors influencing KS development can provide insights into the underlying mechanisms, which creates a worthwhile basis for investigating genetic susceptibility and viral interaction mechanisms that may unveil novel therapeutic targets. Finally, investigating innovative therapeutic interventions, including immunomodulatory strategies, targeted therapies, and potential vaccine development, holds promise for improving treatment outcomes and preventing KS development.
